# Essential Oil-Based Design and Development of Novel Anti-*Candida* Azoles Formulation

**DOI:** 10.3390/molecules25061463

**Published:** 2020-03-24

**Authors:** Rania Hamdy, Bahgat Fayed, Alshaimaa M. Hamoda, Mutasem Rawas-Qalaji, Mohamed Haider, Sameh S. M. Soliman

**Affiliations:** 1 Research Institute for Medical and Health Sciences, University of Sharjah, P.O. Box, Sharjah 27272, UAE; r.badawy@cuca.ae (R.H.); bfayed@sharjah.ac.ae (B.F.); alshaimaahamoda@gmail.com (A.M.H.); mqalaji@sharjah.ac.ae (M.R.-Q.); mhaider@sharjah.ac.ae (M.H.); 2 Department of Medicinal Chemistry, Faculty of Pharmacy, Zagazig University, Zagazig 44519, Egypt; 3 Chemistry of Natural and Microbial Product Department, National Research Centre, Cairo 12622, Egypt; 4 Department of Pharmacognosy, Faculty of Pharmacy, Assiut University, Assiut 71515, Egypt; 5 College of Pharmacy, University of Sharjah, P.O. Box, Sharjah 27272, UAE; 6 Department of Pharmaceutics and Industrial Pharmacy, Faculty of Pharmacy, Cairo University, Cairo 11562, Egypt

**Keywords:** *Candida albicans*, *Candida auris*, anti-fungal, nanoparticles, azoles, essential oil

## Abstract

*Candida* is the most common fungal class, causing both superficial and invasive diseases in humans. Although *Candida albicans* is the most common cause of fungal infections in humans, *C. auris* is a new emergent serious pathogen causing complications similar to those of *C. albicans*. Both *C. albicans* and *C. auris* are associated with high mortality rates, mainly because of their multidrug-resistance patterns against most available antifungal drugs. Although several compounds were designed against *C. albicans*, very few or none were tested on *C. auris*. Therefore, it is urgent to develop novel effective antifungal drugs that can accommodate not only *C. albicans*, but also other *Candida* spp., particularly newly emergent one, including *C. auris*. Inspired by the significant broad-spectrum antifungal activities of the essential oil cuminaldehyde and the reported wide incorporation of azoles in the antifungal drugs, a series of compounds (**UoST1-11**) was designed and developed. The new compounds were designed to overcome the toxicity of the aldehyde group of cuminaldehyde and benefit from the antifungal selectivity of azoles. The new developed UoST compounds showed significant anti-*Candida* activities against both *Candida* species. The best candidate compound, **UoST5**, was further formulated into polymeric nanoparticles (NPs). The new formula, UoST5-NPs, showed similar activities to the nanoparticles-free drug, while providing only 25% release after 24 h, maintainng prolonged activity up to 48 h and affording no toxicity. In conclusion, new azole formulations with significantly enhanced activities against *C. albicans* and *C. auris*, while maintaining prolonged action and no toxicities at lower concentrations, were developed.

## 1. Introduction

*Candida* species are the most common cause of fungal infections in humans. Although *Candida* can cause treatable superficial infections [1], they can also generate serious health problems, particularly to immunocompromised patients [2,3]. *C. auris* is a newly-emerging *Candida* species [4,5]. Both *C. albicans* and *C. auris* are considered as serious global health threats mainly because of their multidrug-resistance patterns associated with high mortality rates that can reach 60% [6,7,8].

The most common antifungal drugs present in the market [9] can be classified into polyene macrolides (amphotericin B and nystatin) that lead to alteration in the fungal membrane, azole derivatives (ketoconazole, fluconazole, itraconazole, and voriconazole) that inhibit 14α-lanosterol demethylase (CYP51A1), a key enzyme in ergosterol biosynthesis, DNA and RNA synthesis inhibitors (flucytosine), and echinocandins (casofungin and micafungin) that inhibit 1,3-β-glucan synthase.

Drug resistance among *Candida* species is one of the greatest challenges for the clinical success of antifungal agents. The mechanisms of *Candida* resistance against antifungal drugs can include up-regulation of the target enzyme, alteration in the target site of the enzyme, increase in drug efflux, permeability barriers associated with biofilm formation, and development of mutations in the ERG3 gene that result in cell membranes devoid of ergosterol [10]. Furthermore, the emerging threats of multidrug-resistant *C. auris* are also alarming. Therefore, there is a growing demand for the development of novel anti-*Candida* agents to overcome the different mechanisms of resistance to the currently available drugs. The aim of this study was thus to develop novel antifungal drugs effective against not only *C. albicans* but ones that can also accommodate other *Candida* spp. including in particular the newly emergent *C. auris*.

Recently, we have reported that cuminaldehyde essential oil isolated from *Calligonum comosum* plant shows excellent antifungal activities [11,12]. Despite its excellent antifungal activities, nevertheless it showed poor bioavailability since it is lipid-soluble and displays very high toxicity to mammalian cells due to its aldehyde nature. Therefore, we have designed a series of novel compounds via structural modification of the natural scaffold by replacing the aldehyde group with a 1,2,4-triazole ring carrying a substituted benzylsulfanyl moiety [13], presumably, to generate new derivatives with enhanced physicochemical properties and safety profiles. The newly designed azoles were formulated into polymeric NPs in order to enhance the compounds’ bioavailability, reduce the toxicity and prolong the activities. 

Formulation of antifungal drugs into polymeric NPs is one of the approaches that have been utilized to overcome poor drug solubility for hydrophobic antifungal drugs It provides an appropriate time for the drug to reach infected sites, and enhance drug release for an extended time to improve patient compliance [14]. Additionally, polymeric NPs can protect the loaded drug from degradation by the acidic pH in the stomach and from liver enzymes after oral administration, therefore, enhancing their bioavailability and absorption compared to the free drug [15,16]. In the present work, poly(lactic-co-glycolic acid) (PLGA) has been employed to encapsulate the new UOST5 triazole derivatives into NPs. The mentioned polymer was selected as it is biocompatible and has been approved by the FDA, in addition to the ability to control the drug release rate by adjusting the PLGA characters such as molecular weight and glycolic acid/lactic acid molar ratio [17]. Hence one can eventually control the degradation rate.

## 2. Results

### 2.1. Rational Sesign of **UoST1-11**, Novel Anti-Candida Compounds Based on an Essential Oil Natural Product Scaffold 

**Figure 1:**
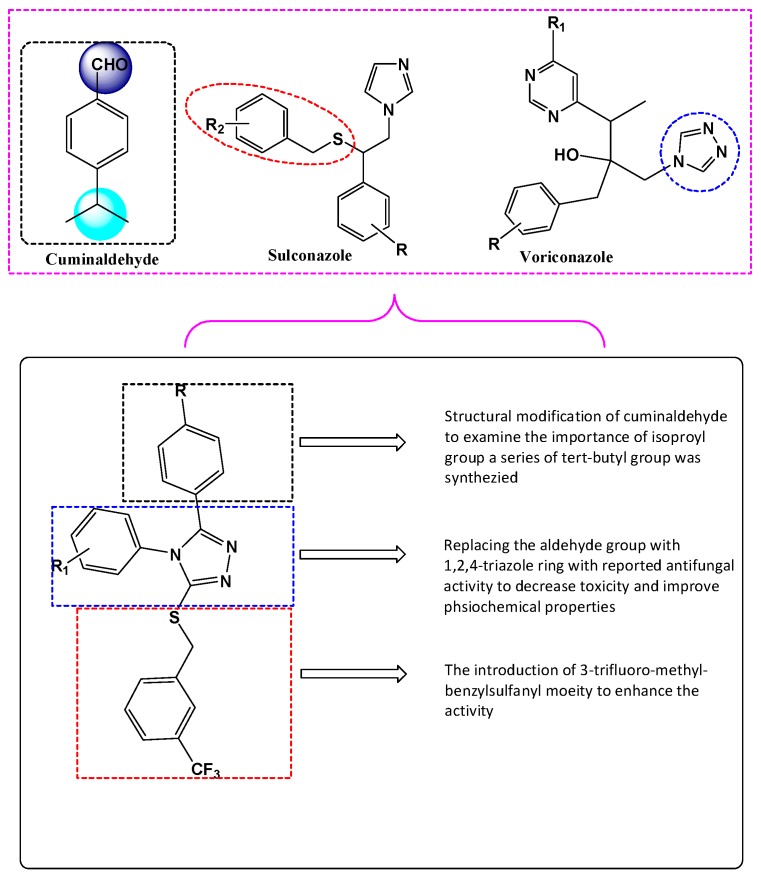
Rational design and synthesis of UoST compounds.

Cuminaldehyde, a natural essential oil, was used as lead structure to develop novel safe and effective anti-*Candida* agents. Inspired by the selective antifungal activity of azoles and the reported antifungal activities of cuminaldehyde [11], a series of UoST compounds was designed and synthesized (Figure 1).

**Scheme 1:**
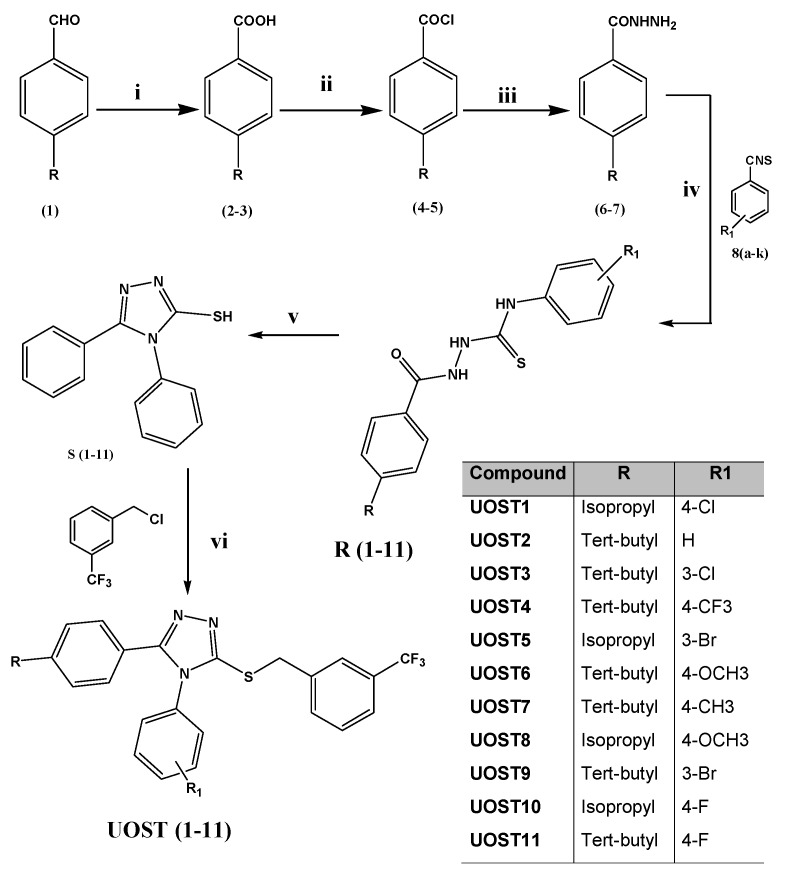
Synthesis of UoST compounds. *Reagents and conditions*: (**i**) NaOH-I2/TBHP, 70 °C, 10–16 h; (**ii**) oxalyl chloride/anhydrous DCM/DMF; (**iii**) NH_2_NH_2_·H_2_O/Ethanol; (**iv**) ethanol/reflux 3 h; (**v**) 2N NaOH/reflux for 3 h; (**vi**) KOH-ethanol-12 h/stirring.

The toxic aldehyde group of cuminaldehyde was replaced by a 1,2,4-triazole ring followed by the introduction of the 3-trifluoromethylbenzylsulfanyl moiety to selectively enhance the antifungal activity and reduce the toxicity. The synthesis of target compounds (**UoST1-11**) was performed as described in Scheme 1.

Initially, cuminaldehyde (**1**) was oxidized to cumnic acid (**2**) by iodine, NaOH and TBHP as catalyst in moderate yield. The acids **2**, **3** were then converted to acid chlorides **4**, **5** using oxalyl chloride, DCM and DMF under nitrogen at 0 °C. The acid hydrazide derivatives **6, 7** were obtained in high yields by refluxing the acid chlorides with hydrazine hydrate [18]. The hydrazides derivatives **6, 7** were treated with substituted phenyl isothiocyanates **8a**–**k** for the synthesis of the corresponding thiosemicarbazides **R (1**–**11)**, which were subsequently converted to triazole thiols **S (1–11)** using 2N NaOH. The target compounds **UoST1**–**11** were formed in moderate yields (~70%) by nucleophilic substitution reactions between triazole thiols **S (1**–**11)** and 3-trifluorobenzyl chloride.

### 2.2. Selective Inhibition Activities of UoST Compounds Against Candida Spp. 

**Figure 2:**
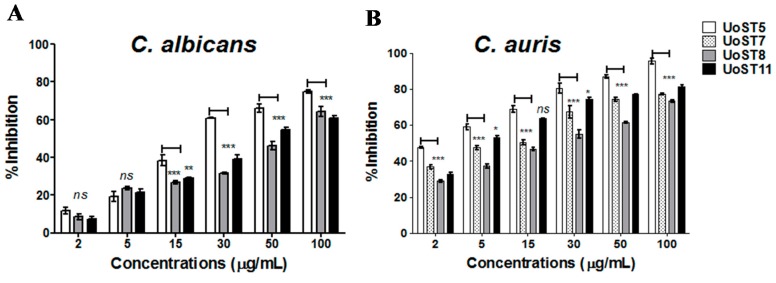
Anti-*Candida* activities of UoST compounds. (**A**) *C. albicans* growth inhibition by **UoST5**, **8** and **11** compounds at different concentrations (2, 5, 15, 30, 50, 100 µg/mL). (**B**) *C. auris* growth inhibition by **UoST5**, **7**, **8** and **11** compounds at different concentrations (2, 5, 15, 30, 50, 100 µg/mL). *Candida* at 10^4^ CFU/mL was grown in 96 well microplate containing YPD broth. The plates were incubated at 37 °C for 24 h prior to measurement at 600 nm using plate reader. Cultures without compounds were employed as negative control. The data was analyzed using two-way ANOVA and statistical significance was calculated with Bonferroni’s multiple comparisons test and significance level indicated by asterisks (* *P* < 0.05; ** *P* < 0.01: *** *P* < 0.001). The data display the mean ± standard error (SEM) of three replicas.

The anti-*Candida* activities of the developed compounds **UoST1**–**11** were tested against *C. albicans* and *C. auris*. The results obtained indicated that compounds **UoST5**, **7**, **8** and **11** caused significant variable inhibition activities on the growth of both *C. albicans* (Figure 2A) and *C. auris* (Figure 2B) at different concentrations (2, 5, 15, 30, 50 and 100 µg/mL) when compared to negative control. Compound **UoST5** showed persistent and significant promising activities on both *Candida* spp. when compared to other compounds (two-way ANOVA, *P* < 0.0001, Figure 2) and with the lowest MIC_50_ (2 µg/mL) on *C. auris* when compared to amphotericin B, employed as positive control (Table 1). On the other hand, compound **UoST7** showed selective inhibition activities on *C. auris* but not *C. albicans* (Figure 2). 

### 2.3. Formulation and Characterization of **UoST5**-Loaded NPs

**Figure 3:**
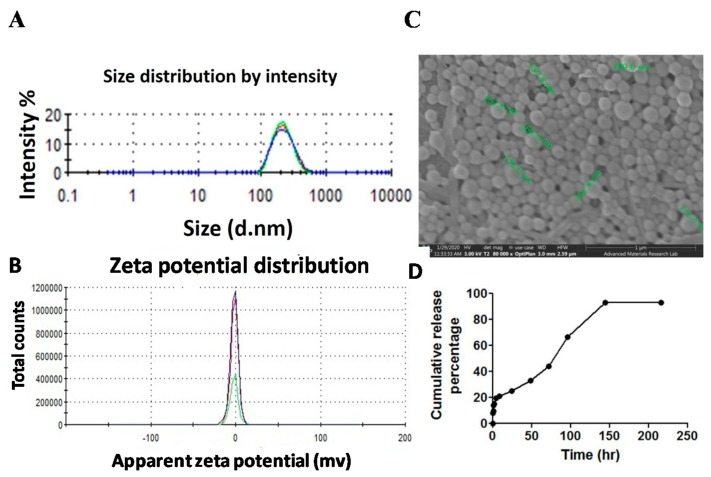
Characterization of nanoparticles. (**A**,**B**) Particle size distribution and zeta potential measurement using a Malvern Instruments Zetasizer Nano ZS. NPs samples were diluted with distilled water and the polydispersity index (**A**) and zeta potential (**B**) were determined using the Dynamic light scattering (DLS) mode and Laser Doppler Velocimetry (LDV) mode, respectively. The measurement was performed in triplicate. (**C**) SEM micrographs of the developed nanoparticles. The NPs samples after complete dryness were placed on standard carbon tape and coated with gold prior to measurement. (**D**) Release pattern of UoST5-loaded NPs. UoST5-NPs were placed into dialysis membrane (MWCO = 12 kDa) containing 1ml of release media (0.9% NaCl and isopropanol (85:15), then immersed in 10 mL of release media and incubated in shaking water bath at 37 °C and 50 rpm.

Compound **UoST5** was formulated into PLGA NPs using the emulsion/evaporation method. The size and shape of the developed NPs were analyzed by a Zeta analyzer and scanning electron microscope (SEM), respectively. The particle size and size distribution were evaluated by a Zetasizer and found to be 199.5 ± 0.035 nm with narrow size distribution (0.098 ± 0.016) (Figure 3A). Additionally, the particle surface charge (Zeta potential) was −1.58 ± 0.25 mV (Figure 3B). The surface morphology and topography of UoST5-loaded NPs showed well-defined spherical shaped NPs with smooth surfaces without any cracks (Figure 3C). The amount of drug entrapped was calculated directly by dissolving the NPs in DCM. The drug release was measured by UV/VIS spectrophotometer and the entrapment efficiency was calculated to be 67.2%.

The release of **UoST5** from PLGA NPs was determined within 10 days by incubating certain amount of UoST5-NPs in a release media at 37 °C followed by measurement at different time intervals. The release pattern was biphasic with an initial burst release of 19.79% after 4 h (Figure 3D). This was followed by a gradual release as the cumulative release percentage reached 25.039% after 24 h and 92.9% after 6 days and stayed at the same percentage for another 4 days (Figure 3D). 

### 2.4. NP Formulation Enhanced and Prolonged the Anti-Candida Activities of **UoST5**

**Figure 4:**
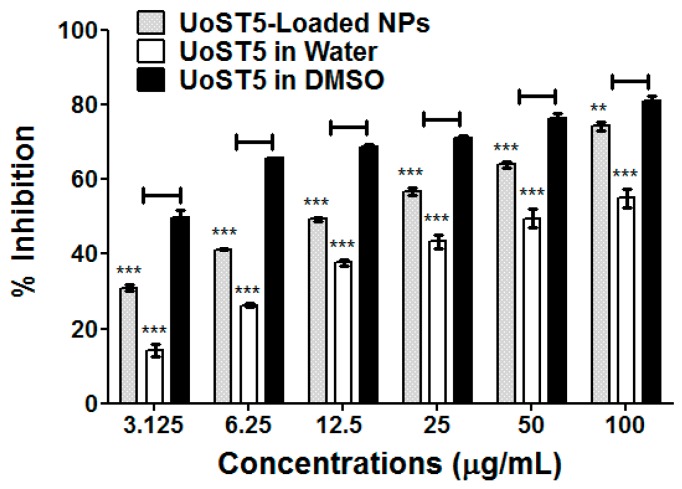
Anti-*Candida* activity of UoST5-Loaded NPs compared to free synthesized compound. The anti-*Candida auris* activity of UoST5-NPs in water was compared to UoST5 in water and DMSO at different concentrations (3.125, 6.25, 12.5, 25, 50 and 100 µg/mL). *C. auris* at 10^4^ CFU/mL was grown in 96 well microplate containing YPD broth. The plates were incubated at 37 °C for 24 h prior to measurement at 600 nm using a plate reader. Cultures without compounds were employed as negative control. The data display the mean of the percentage of the survival rate of mammalian cells ± standard error of the mean. A *P*-value < 0.05 was considered as significant. The level of significance was indicated by asterisks (** *P* < 0.01: *** *P* < 0.001)

The bioactivity of **UoST5**-loaded NPs on *C. auris* was compared to the free drug (suspended in water or dissolved in DMSO). The results showed that UoST5-NPs (suspended in water) achieved significantly higher activity than when the compound was suspended in water but lower than when dissolved in DMSO (two-way ANOVA, *P* < 0.0001, Figure 4). The anti-*Candida* activities of UoST5-NPs were comparable to the compound in DMSO (Figure 4), while afforded no toxicity to mammalian cells at six times the MIC_50_ concentrations (Data not shown). Drug-free NPs, employed as control, showed no effects on *Candida* or mammalian cells.

**Figure 5:**
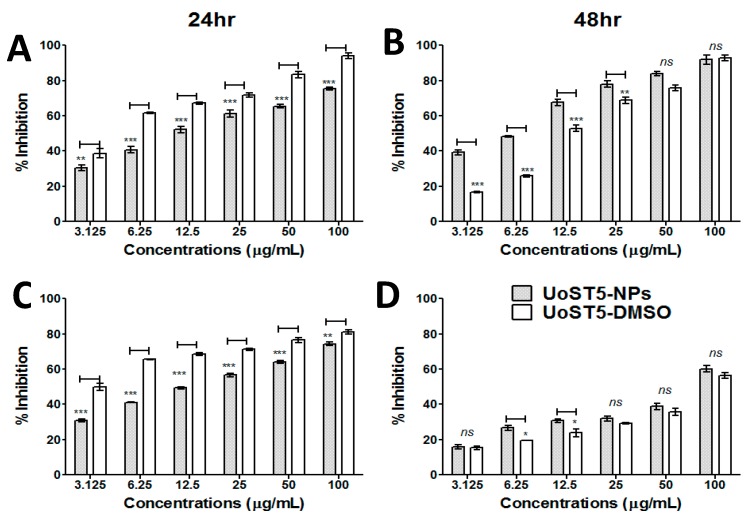
Anti-*Candida* activities of UoST5-NPs compared to free UoST5 at (**A**,**C**) 24 h and (**B**,**D**) 48 h. (**A**,**B**) represent the effects of the drugs on *C. albicans*, while (**C**,**D**) represent the effects of the drugs on *C. auris*. *Candida* growth inhibition was measured at different concentrations (3.125, 6.25, 12.5, 25, 50 and 100 µg/mL). *Candida* at 10^4^ CFU/mL was grown in 96 well microplate containing YPD broth. The plates were incubated at 37 °C for 24 h prior to measurement at 600 nm using plate reader. Cultures without compounds were employed as negative control in order to measure percent inhibition. The data was analyzed using two-way ANOVA and statistical significance was calculated with Bonferroni’s multiple comparisons test and significance level indicated by asterisks (* *P* < 0.05; ** *P* < 0.01: *** *P* < 0.001). The data display the mean ± standard error (SEM) of three replicates.

The anti-*Candida* activities of UoST5-loaded NPs were compared to NPs-free drug dissolved in DMSO at concentrations range 3.12–100 µg/mL within 48 h (Figure 5). Both the free drug and NPs formulations exhibited significant growth inhibition activity on both *C. albicans* and *C. auris* with different magnitudes depending on the drug concentrations and time of incubation. In the case of *C. albicans*, the inhibition effect of UoST5-NPs after 24 h incubation was 75.46% at 100 µg/mL which is significantly lower than the effect of free drug (two-way ANOVA, *P* < 0.0001, Figure 5A). The inhibition was further enhanced after 48 h to reach 91.8% similarity to the effect of free drug (Figure 5B). At 3.12 µg/mL, the inhibition effect was 30.58%, and 39.3% when compared to free drug (40% and 19%, two-way ANOVA, *P* < 0.0001) after 24 h and 48 h, respectively. A similar pattern was obtained in the case of *C. auris* (Figure 5C,D). UoST5-NPs at 100 and 3.12µg/ml after 24 h showed 74.34% and 30.96% inhibition activity, respectively. Unlike the effect on *C. albicans*, the inhibition effect of UoST5-NPs was slightly reduced after 48 h to reach 60.2% and 15.96% at 100 and 3.12 µg/mL, respectively (Figure 5C,D). Comparing the anti-*Candida* activities of UoST5-loaded NPs to free **UoST5** drug at the same concentration indicated that UoST5-loaded NPs showed lower inhibition activity at 24 h and higher inhibition activity at 48 h (Figure 5), although the released amount of the drug from the NPs was only 25%; thus providing a significant increase in activity. Collectively, we have developed novel triazole derivatives effective against both *C. albicans* and *C. auris* and its formulation in NPs enhanced and prolonged the activities of the drug.

## 3. Discussion

*Candida* spp. are responsible for 75–88% of all fungal infections in the US, causing an extra annual health cost of $1.7 billion [19]. The general resistance of *Candida* spp. to most available antifungal drugs [20] and the emergence of multidrug resistant *C. auris* [21] make urgent the need to develop novel effective anti-*Candida* drugs. On the other hand, most compounds synthesized nowadays are developed against *C. albicans*, while very few are tested against *C. auris*. Thus, we proposed to develop novel azoles derivatives with effective antifungal activities against *Candida* spp. including in particular *C. auris*. Azole derivatives show great potential activities against *Candida* infections. Chandrika et al. developed 12 novel azole derivatives that showed significant antifungal activities against different types of *Candida* spp., but none of them was *C. auris* [22]. Similarly, Shrestha et al., successfully developed some novel alkylated-azoles that showed great activities against 13 clinically fungal isolates, but *C. auris* was not one of them [23]. Here, we have developed a series of novel S-alkylated azole derivatives (compounds **UoST1**–**11**) by cyclization reactions of thiosemicarbazide intermediates **R**(**1**–**11**) followed by S-alkylation in alkaline media. 

Generally, 1,2,4-triazole-3-thiones have attracted considerable attention because of their broad biological activities [24]. Some of our developed compounds (compounds **UoST5**, **7**, **8** and **11**) showed variable promising activities against both *C. albicans* and *C. auris*. The variability of the activity is attributed to the variation in the substitution on the newly developed structures including the isopropyl or *tert*-butyl substituents at the C-4 position of the phenyl ring (R) and the substituted phenyl attached to the N-4 position of the triazole ring (R1) carrying 3-Cl, 4-Cl, 4-CH_3_, 4-OCH_3_, 4-F, 3-Br or unsubstituted phenyl. Looking at the chemical structures of the most active compounds **UoST5**, **7**, **8** and **11** that showed significant anti-*Candida* activity, it can be declared that 3-Br (**UoST5**), 4-OCH_3_ (**UoST8**) substitution on R1 with an isopropyl substituent on R, and the *tert*-butyl substituent on R with 4-F on R1 (**UoST11**) are active against both *Candida* spp., while the substitution with 4-CH_3_ on R1 with *tert*-butyl on R (**UoST7**) was significantly selective against *C. auris*.

Compared to the positive control, **UoST5** was selected as candidate for further development mainly because of its significant anti-*Candida* activities against both *Candida* spp., its low MIC_50_ value and its safety on mammalian cells. Like 90% of the molecules in the discovery pipeline, **UoST5** showed limited aqueous solubility that consequently leads to poor bioavailability [25]. In view of the foregoing, we have enhanced the bioavailability of **UoST5** by encapsulation into PLGA-NPs. The single emulsion solvent evaporation method was employed for UoST5-NPs fabrication [26]. The aforementioned fabrication method of hydrophobic drugs affords simplicity with the ability to scale up [26,27,28]. The obtained drug entrapment efficiency of 67.2% is similar to other reports using the same method [14,29]. The relatively higher entrapment efficiency reported here is one of the main advantages of encapsulating hydrophobic drugs into PLGA-NPs. This can be attributed to the hydrophobic nature of the biodegradable polymer (PLGA) employed [15,26]. Furthermore, a relatively uniform particle distribution was obtained as the polydispersity index value was 0.098. The relatively low particle charge obtained was also favorable since it increases the contact and interaction of the NPs with the *Candida* outer membrane. Generally, *Candida* carry negative surface charge; thus it was essential to develop NPs that carry either neutral or low negative charge to avoid any repulsion forces that may prevent the attachment of NPs to the *Candida* surface [30,31]. The release of **UoST5** from the NPs followed the common biphasic pattern with an initial burst release followed by sustained release of the drug over 6 days. The initial burst release of the drug is mainly related to the fraction of the drug located and adsorbed on the surface of the NPs that has been reported and described elsewhere [32,33]. The sustained release is relevant to the amount of the drug encapsulated and the degradation rate of PLGA employed. 

**Figure 6:**
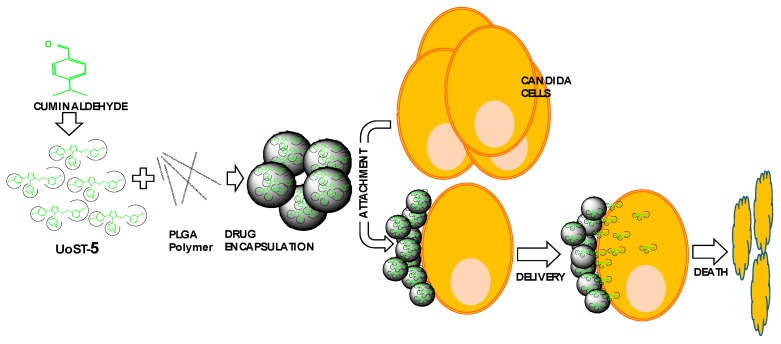
Graphical representation summarizes the synthesis, formulation and proposed mechanism of attachment and delivery of UoST5-NPs.

The bioactivity of UoST5-NPs compared to the free **UoST5** can be explained by the UoST5-NPs’ release pattern. Although 25% of the entrapped drug was released from the NPs after 24 h, the inhibition effects on *C. albicans* and *C. auris* were 62.8–91.7% of the inhibition effect obtained by the free drug (100% in direct contact with the *Candida* cells). Interestingly, **UoST5** release after 48 h reached up to 32.95%, while exerting similar or higher activities on *Candida* cells when compared to 100% free drug. Initially, this confirms that the method and reagents used to encapsulate **UoST5** did not modify or degrade the **UoST5** molecule as the drug conserved its bioactivity. Additionally, as the PLGA-NPs free of UoST5 has no inhibitory effect on *Candida*, the enhancement of the antifungal activity of UoST-NPs can be related to the ability of the NPs to adhere and attach to the outer surface of *Candida* cells. Therefore, it provides direct contact and reduces the loss of the drug. The ability of the NPs to adhere and even infiltrate to fungal cells was previously studied and explained [14,34]. A model represents the synthesis of NPs, attachment to *Candida* cells and delivery of UoST5 is described in Figure 6. Essential oils including cuminaldehyde are known for their promising antimicrobial activities [35], while inducing toxic effects on human cells [11]. Therefore, extraction and purification of essential oil followed by few synthetic steps in order to optimize its selective antimicrobial activities and provide no toxicity will be of great value for both human and market uses.

## 4. Materials and Methods

### 4.1. Materials

Most chemicals and solvents were of analytical grade and, when necessary, were purified and dried by standard methods. Poly (D,L-lactic-co-glycolic acid; PLGA) (50/50, M.W. 24–38 kDa), polyvinyl alcohol (PVA), with molecular weight of MW 13,000–23,000, 87–89% hydrolyzed were purchased from Sigma-Aldrich (St. Louis, MO, USA). Dichloromethane (DCM) was purchased from Merck (Darmstadt, Germany).

### 4.2. General Information

Reactions were monitored by thin-layer chromatography (TLC) using pre-coated silica gel plates (Kieselgel 60 F254, BDH, Taufkirchen, Germany), and spots were visualized under UV light (254 nm). Melting points (mp) were determined using a Gallenkamp melting point apparatus (London, UK). ^1^H-NMR and ^13^C-NMR spectra were recorded at 500/125 MHz on a Bruker spectrometer (Karlsruhe, Germany). Chemical shifts were expressed in parts per million (ppm) relative to tetramethylsilane, and coupling constant (J) values were represented in Hertz (Hz) and the signals were designated as follows: s, singlet; d, doublet; t, triplet; m, multiplet. Mass spectroscopic data were obtained using electrospray ionization (ESI) mass spectroscopy (Bruker Daltonics mass spectrometer, Bremen, Germany).

#### 4.2.1. Synthesis of 4-Isopropyl Benzoic Acid (Cuminic Acid) (**2**)

Cuminic acid (**2**) was prepared by the oxidation of cuminaldehyde (**1**) following a reported procedure [36]. Cuminaldehyde (10 mmol) in water was mixed with iodine and NaOH (10 mmol) and TBHP (20 mmol) as catalyst [36]. Yield 62%. ^1^H-NMR (DMSO-d_6_): δ 1.21 (d, 6H, 2CH_3_ of isopropyl), 2.90 (m, 1H, CH of isopropyl), 7.34 (d, 2H, *J* = 8, ArH), 7.52 (d, 2H, *J* = 7.5, ArH).

#### 4.2.2. General Procedure for the Synthesis of 4-Substituted Benzoyl Chlorides **4**, **5**

Oxalyl chloride (12 mmol) was added dropwise to a mixture of the appropriate 4-substituted benzoic acid **2, 3** (10 mmol), in anhydrous DCM (30 mL) and anhydrous DMF (10 µL) under nitrogen at 0 °C. The reaction mixture was stirred at room temperature for sn additional 3 h until the reaction was completed. The reaction mixture was evaporated under vacuum and the oily residue was used without further purification for the acid hydrazide preparation.

#### 4.2.3. General Procedure for the Synthesis of 4-Substituted Benzoic Acid Hydrazides **6**, **7**

The synthesis of 4-substituted benzoic acid hydrazides was performed as previously described [18]. To a solution of ethyl 4-substituted benzoyl chloride **4**, **5** (10 mmol) in ethanol (10 mL), excess hydrazine monohydrate (5 mL) was added. The reaction mixture was refluxed for 24 h, and left to cool to room temperature. The formed precipitate was collected by filtration, washed with water followed by cold ethanol to remove excess hydrazine and left to dry to give the corresponding acid hydrazide.

*4-Isopropyl-benzoic acid hydrazide* (**6**). Yield 85%, ^1^H-NMR (DMSO-d_6_): δ 1.21 (d, 6H, 2 CH_3_ of isopropyl), 2.93 (m, 1H, CH of isopropyl), 4.45 (s, 2H, NH_2_), 7.31 (d, 2H, *J* = 8, ArH), 7.54 (d, 2H, *J* = 6.5, ArH), 9.67 (s, 1H, NH).

*4-tert-Butyl-benzoic acid hydrazide* (**7**). Yield 87%, ^1^H-NMR (DMSO-d_6_): δ 1.29 (s, 9H, *tert*-butyl), 4.45 (s, 2H, NH_2_), 7.45 (d, 2H, *J* = 8.5, ArH), 7.75 (d, 2H, *J* = 6.5, ArH), 9.68 (s, 1H, NH).

#### 4.2.4. General Procedure for Preparation of 4-Substituted Benzoyl-*N*-Substituted Phenyl Thiosemicarbazides **R**(**1**–**10**)

The 4-substituted benzoyl-*N*-substituted phenyl thiosemicarbazide **R** derivatives were synthesized as previously described [18]. To a solution of 4-substituted benzoic acid hydrazide **6, 7** (10 mmol) in ethanol (30 mL) a solution of substituted phenyl isothiocyanate **8a**–**k** (10 mmol) in ethanol (20 mL) was added with continuous stirring. The reaction mixture was refluxed for 3 h. After being cooled to room temperature, the precipitate formed was collected by filtration, washed with cold ethanol (30 mL) to give the corresponding thiosemicarbazide **R**(**1**–**10**)**,** Yield 79%.

*4-Isopropyl-benzoyl-N-(4-chlorophenyl) hydrazine carbothioamide* (**R1**). Yield 75%, ^1^H-NMR (DMSO-d_6_): δ 1.21 (d, 6H, 2CH_3_ of isopropyl), 2.95 (m, 1H, CH of isopropyl), 7.38 (d, 4H, *J* = 8, ArH), 7.48 (d, 2H, *J* = 6.5, ArH), 9.88 (d, 2H, *J* = 8, ArH), 9.7 (s, 2H, NH), 10.47 (s, 1H, NH).

*4-tert-Butyl-benzoyl-N-(phenyl) hydrazine carbothioamide* (**R2**). Yield 70%, ^1^H-NMR (DMSO-d_6_): δ 1.31 (s, 9H, *tert*-butyl), 7.15 (t, 1H, *J* = 7.5, ArH), 7.32 (t, 2H, *J*= 7.5, ArH), 7.34 (d, 2H, *J* = 6.5, ArH), 7.52 (d, 2H, *J* = 2, ArH), 7.90 (d, 2H, *J* = 8, ArH), 9.6 (s, 1H, NH), 9.76 (s, 1H, NH), 10.46 (s, 1H, NH).

*4-tert-Butyl-benzoyl-N-(3-chlorophenyl) hydrazine carbothioamide* (**R3**). Yield 64%, ^1^H NMR (DMSO-d_6_): δ 1.31 (s, 9H, *tert*-butyl), 7.2 (d, 1H, *J* = 7.5, ArH), 7.35 (t, 1H, *J* = 8, ArH), 7.48 (d, 1H, *J* = 7, ArH), 7.53 (d, 2H, *J* = 7, ArH), 7.61 (s, 1H, ArH), 7.90 (d, 2H, *J* = 8.5, ArH), 9.83 (s, 1H, NH), 9.89 (s, 1H, NH), 10.50 (s, 1H, NH).

*4-tert-Butyl-benzoyl-N-(4-trifluoromethylphenyl) hydrazine carbothioamide* (**R4**). Yield 62%, ^1^H-NMR (DMSO-d_6_): δ 1.31 (s, 9H, *tert*-butyl), 7.52 (d, 2H, *J* = 7, ArH), 7.68 (d, 2H, *J* = 8.5, ArH), 7.76 (d, 2H, *J* = 2, ArH), 7.90 (d, 2H, *J* = 8.5, ArH), 9.96 (s, 2H, NH), 10.53 (s, 1H, NH).

*4-Isopropyl-benzoyl-N-(3-bromophenyl) hydrazine carbothioamide* (**R5**). Yield 70%, ^1^H-NMR (DMSO-d_6_): δ 1.23 (d, 6H, 2 CH_3_ of isopropyl), 2.94 (m, 1H, CH of isopropyl), 7.31 (m, 2H, *J* = 8, ArH), 7.32 (d, 1H, *J* = 4, ArH), 7.39 (d, 1H, *J* = 8.5, ArH), 7.52 (d, 1H, *J* = 8.5, ArH), 7.72 (s, 1H, ArH), 7.89 (d, 2H, *J* = 8, ArH), 9.83 (s, 1H, NH), 9.87 (s, 1H, NH), 10.49 (s, 1H, NH).

*4-tert-Butyl-benzoyl-N-(4-methoxyphenyl) hydrazine carbothioamide* (**R6**). Yield 64%, ^1^H-NMR (DMSO-d_6_): δ 1.31 (s, 9H, *tert*-butyl), 3.75 (s, 3H, OCH_3_), 6.89 (d, 2H, *J* = 9, ArH), 7.28 (d, 2H, *J* = 6.5, ArH), 7.50 (d, 2H, *J* = 4.5, ArH), 7.89 (d, 2H, *J* = 6, ArH), 9.58 (s, 1H, NH), 9.66 (s, 1H, NH), 10.43 (s, 1H, NH).

*4-tert-Butyl-benzoyl-N-(p-tolyl) hydrazine carbothioamide* (**R7**). Yield 69%, ^1^H-NMR (DMSO-d_6_): δ 1.31 (s, 9H, *tert*-butyl), 2.28 (s, 3H, CH_3_), 7.12 (d, 2H, *J* = 8.5 ArH), 7.31 (d, 2H, *J* = 6.5, ArH), 7.51 (d, 2H, *J* = 8.5, ArH), 7.89 (d, 2H, *J* = 8, ArH), 9.62 (s, 1H, NH), 9.69 (s, 1H, NH), 10.44 (s, 1H, NH).

*4-Isopropyl-benzoyl-N-(3-methoxyphenyl) hydrazine carbothioamide* (**R8**). Yield 51%, ^1^H-NMR (DMSO-d_6_): δ 1.12 (d, 6H, 2 CH_3_ of isopropyl), 2.93 (m, 1H, CH of isopropyl), 3.68 (s, 3H, OCH_3_), 6.72 (dd, 1H, *J*= 8, ArH), 7.02 (d, 1H, *J* = 8, ArH), 7.10 (s, 1H, ArH), 7.10 (t, 1H, *J* = 8.5, ArH), 7.24 (d, 2H, *J* = 8, ArH), 7.85 (d, 2H, *J* = 8, ArH), 9.65 (s, 2H, NH), 10.43 (s, 1H, NH).

*4-tert-Butyl-benzoyl-N-(3-bromophenyl) hydrazine carbothioamide* (**R9**). Yield 64%, ^1^H-NMR (DMSO-d_6_): δ 1.31 (s, 9H, *tert*-butyl), 6.64 (dd, 2H, *J* = 6.5, ArH), 6.98 (d, 1H, *J* = 6, ArH), 7.29 (d, 2H, *J* = 8.5, ArH), 7.48 (d, 1H, *J*= 8, ArH), 7.59 (d, 1H, *J* = 7, ArH), 7.82 (s, 1H, ArH), 9.68 (s, 1H, NH), 9.86 (s, 1H, NH), 10.50 (s, 1H, NH).

*4-Isopropyl-benzoyl-N-(4-fluorophenyl) hydrazine carbothioamide* (**R10**). Yield 63 %, ^1^H-NMR (DMSO-d_6_): δ 1.23 (d, 6H, 2 CH_3_ of isopropyl), 2.97 (m, 1H, CH of isopropyl), 7.37 (dd, 2H, *J* = 8, ArH), 7.68 (d, 2H, *J* = 8.5, ArH), 7.74 (d, 2H, *J* = 6.5, ArH), 7.88 (d, 2H, *J* = 8, ArH), 9.74 (s, 1H, NH), 9.77 (s, 1H, NH), 10.45 (s, 1H, NH).

*4-tert-Butyl-benzoyl-N-(4-fluorophenyl) hydrazine carbothioamide* (**R11**). Yield 64%, ^1^H-NMR (DMSO-d_6_): δ 1.31 (s, 9H, *tert*-butyl), 7.16 (t, 2H, *J* = 9, ArH), 7.42 (d, 2H, *J* = 7, ArH), 7.53 (d, 2H, *J* = 5.5, ArH), 7.90 (d, 2H, *J* = 8.5, ArH), 9.73 (s, 1H, NH), 9.77 (s, 1H, NH), 10.47 (s, 1H, NH).

#### 4.2.5. General Procedure for Synthesis of Triazole Thiols **S**(**1**–**11**)

A solution of 4-substituted benzoyl-*N*-substituted phenyl thiosemicarbazides (10 mmol) **R**(**1**–**11**) in 2 N NaOH (20 mL) were refluxed for 3 h. After cooling to room temperature, water was added and the mixture was carefully neutralized with dilute HCl. The precipitate formed was filtered, dried and re-crystallized from ethanol to give the corresponding triazole thiol, Yield 86%.

*4-(4-Chlorophenyl)-5-(4-isopropylphenyl)-4H-[1,2,4]triazole-3-thiol* (**S1**). Yield 76%, ^1^H-NMR (DMSO-d_6_): δ 1.16 (d, 6H, 2CH_3_ of isopropyl), 2.87 (m, 1H, CH of isopropyl), 7.2 (d, 4H, *J* = 9, ArH), 7.42 (d, 2H, *J* = 5.5, ArH), 7.58 (d, 2H, *J* = 5.5, ArH), 14.10 (s, 1H, SH).

*5-(4-tert-Butylphenyl)-4-phenyl-4H-[1,2,4]triazole-3-thiol* (**S2**). Yield 85%, ^1^H-NMR (DMSO-d_6_): δ 1.22 (s, 9H, *tert*-butyl), 7.22 (d, 2H, *J* = 8.5, ArH), 7.36 (m, 4H, *J* = 9.5, ArH), 7.53 (m, 3H, *J* = 10, ArH), 14.09 (s, 1H, SH).

*5-(4-tert-Butylphenyl)-4-(3-chlorophenyl)-4H-[1,2,4]triazole-3-thiol* (**S3**), Yield 73%, ^1^H-NMR (DMSO-d_6_): δ 1.23 (s, 9H, *tert*-butyl), 7.24 (d, 2H, *J* = 6, ArH), 7.38 (d, 2H, *J* = 5.5, ArH), 7.64 (d, 2H, *J* = 8.5, ArH), 7.92 (d, 2H, *J* = 8, ArH), 14.14 (s, 1H, SH).

*5-(4-tert-Butylphenyl)-4-(4-trifluoromethylphenyl)-4H-[1,2,4]triazole-3-thiol* (**S4**). Yield 72%, ^1^H-NMR (DMSO-d_6_): δ 1.23 (s, 9H, tert-butyl), 7.25 (d, 2H, *J* = 9.5, ArH), 7.34 (d, 1H, *J* = 6.5, ArH), 7.39 (d, 2H, *J* = 6.5, ArH),7.55 (t, 1H, *J* = 8, ArH), 7.60 (m, 2H, *J* = 9, ArH), 14.20 (s, 1H, SH).

*5-(4-Isopropylphenyl)-4-(4-trifluoromethylphenyl)-4H-[1,2,4]triazole-3-thiol* (**S5**). Yield 72%, ^1^H-NMR (DMSO-d_6_): δ 1.21 (d, 6H, 2 CH_3_ of isopropyl), 2.94 (m, 1H, CH of isopropyl), 7.3 (m, 4H, *J* = 12, ArH), 7.46 (d, 2H, *J* = 7.5, ArH), 7.85 (d, 2H, *J* = 8, ArH), 14.20 (s, 1H, SH).

*5-(4-tert-Butylphenyl)-4-(4-methoxyphenyl)-4H-[1,2,4]triazole-3-thiol* (**S6**). Yield 65%, ^1^H-NMR (DMSO-d_6_): δ 1.22 (s, 9H, *tert*-butyl), 3.76 (s, 3H, OCH_3_), 7.24 (d, 2H, *J* = 6.5, ArH), 7.32 (d, 2H, *J* = 8.5, ArH), 7.43 (t, 1H, *J* = 9, ArH) 7.54 (d, 2H, *J* = 5, ArH), 7.67 (s, 1H, ArH), 14.0 (s, 1H, SH).

*5-(4-tert-Butylphenyl)-4-p-tolyl-4H-[1,2,4]triazole-3-thiol* (**S7**). Yield 68%, ^1^H-NMR (DMSO-d_6_): δ 1.22 (s, 9H, *tert*-butyl), 2.37 (s, 3H, CH_3_), 7.24 (m, 4H, *J* = 8.25, ArH), 7.32 (d, 2H, *J* = 8, ArH), 7.37 (d, 2H, *J* = 8.5, ArH), 14.0 (s, 1H, SH).

*5-(4-Isopropylphenyl)-4-(3-methoxyphenyl)-4H-[1,2,4]triazole-3-thiol* (**S8**). Yield 68%, ^1^H-NMR (DMSO-d_6_): δ 1.12 (d, 6H, 2 CH_3_ of isopropyl), 2.82 (m, 1H, CH of isopropyl), 3.70 (s, 3H, OCH_3_), 6.8 (d, 1H, *J* = 6.5, ArH), 6.91 (t, 1H, *J* = 4.5, ArH), 7.06 (dd, 1H, *J* = 8.5, ArH), 7.20 (d, 2H, *J* = 8, ArH), 7.25 (d, 2H, *J* = 8, ArH), 7.39 (t, 1H, *J* = 9.5, ArH), 14.09 (s, 1H, SH). 

*5-(4-tert-Butylphenyl)-4-(3-bromophenyl)-4H-[1,2,4]triazole-3-thiol* (**S9**). Yield 55%, ^1^H-NMR (DMSO-d_6_): δ 1.22 (s, 9H, *tert*-butyl), 7.21 (d, 1H, *J* = 9, ArH), 7.33 (t, 1H, *J* = 8, ArH), 7.32 (d, 1H, *J* = 4, ArH), 7.60 (d, 2H, *J* = 8.5, ArH), 7.83 (d, 2H, *J* = 8.5, ArH), 7.91 (s, 1H, ArH), 14.0 (s, 1H, SH).

*4-(4-Fluorophenyl)-5-(4-isopropylphenyl)-4H-[1,2,4]triazole-3-thiol* (**S10**). Yield 64%, ^1^H-NMR (DMSO-d_6_): δ 1.16 (d, 6H, 2 CH_3_ of isopropyl), 2.87 (m, 1H, CH of isopropyl),), 7.2 (m, 4H, *J* = 9, ArH), 7.42 (d, 2H, *J* = 5.5, ArH), 7.58 (d, 2H, *J* = 5.5, ArH), 14.10 (s, 1H, SH).

*5-(4-tert-Butylphenyl)-4-(4-fluorophenyl)-4H-[1,2,4]triazole-3-thiol* (**S11**). Yield 59%, ^1^H-NMR (DMSO-d_6_): δ 1.22 (s, 9H, *tert*-butyl), 7.25 (d, 2H, *J* = 6.5, ArH), 7.36 (m, 4H, *J* = 12, ArH), 7.44 (m, 2H, *J* = 12, ArH), 14.11 (s, 1H, SH).

#### 4.2.6. General Procedure for S-Alkylated Triazole Thiols **UoST 1**–**11**

To a solution of triazole thiol **S (1-11)** (10 mmol) in 70 % ethanol containing KOH (10 mmol), a solution of 3-trifluorobenzyl chloride (10 mmol) in ethanol was added dropwise. The reaction mixture was left with stirring at room temperature for 16 h. The formed precipitate was collected by filtration and re-crystallized from ethanol to give the pure compounds **UoST 1**–**11**.

*4-(4-Chlorophenyl)-3-(4-isopropylphenyl)-5-(3-trifluoromethylbenzylsulfanyl)-4H-[1,2,4]triazole* (**UoST1**). Yield 60%, mp: 210–212 °C, ^1^H-NMR (DMSO-d_6_): δ 1.15 (d, 6H, 2 CH_3_ of isopropyl), 2.85 (m, 1H, CH of isopropyl), 4.44 (s, 2H, CH_2_), 7.23 (d, 2H, *J* = 8.5, ArH), 7.25 (d, 2H, *J* = 2.5, ArH), 7.26 (d, 2H, *J* = 2, ArH), 7.54 (m, 2H, *J* = 9.5, ArH), 7.56 (d, 1H, *J* = 2, ArH), 7.62 (m, 2H, *J* = 9, ArH), 7.64 (s, 1H, ArH). ^13^C-NMR (DMSO-d_6_): δ 23.71, 33.39, 36.19, 123.97, 124.38, 124.41, 125.35, 125.68, 125.71, 126.89, 128.13, 129.19, 129.44, 129.76, 129.77, 130.14, 132.89, 133.33, 134.90, 139.17, 150.69, 151.05, 154.75. MS analysis for C_25_H_21_ClF_3_N_3_S: Calcd mass: 487.97, found (*m*/*z*, ES+): 488.27

*3-(4-tert-Butylphenyl)-4-phenyl-5-(3-trifluoromethylbenzylsulfanyl)-4H-[1,2,4]triazole* (**UoST2**). Yield 70%, mp: 198–200 °C. ^1^H-NMR (DMSO-d_6_): δ 1.22 (s, 9H, *tert*-butyl), 4.46 (s, 2H, CH_2_), 7.24 (m, 3H, *J* = 8.25, ArH), 7.34 (d, 2H, *J* = 8, ArH), 7.51 (m, 4H, *J* = 8.5, ArH), 7.63 (t, 2H, *J* = 9.5, ArH), 7.70 (s, 1H, ArH). ^13^C-NMR (DMSO-d_6_): δ 30.95, 34.62, 35.90, 120.97, 123.13, 123.81, 124.29, 125.50, 125. 56, 125.63, 125.67, 127.67, 127.77, 128.86, 129.12, 129.37, 129.65, 130.25, 133.28, 133.97, 139.18, 151.12, 152.69, 154.53. MS analysis for C_26_H_24_F_3_N_3_S: Calcd mass: 467.16, found (*m*/*z*, ES+): 468.23.

*3-(4-tert-Butylphenyl)-4-(3-chlorophenyl)-5-(3-trifluoromethylbenzylsulfanyl)-4H-[1,2,4]triazole* (**UoST3**). Yield 70%, mp: 230–232 °C, ^1^H-NMR (DMSO-d_6_): δ 1.23 (s, 9H, *tert*-butyl), 4.45 (s, 2H, CH_2_), 7.21 (dd, 1H, *J* = 2, ArH), 7.28 (dd, 2H, *J* = 2, ArH), 7.38 (dd, 2H, *J* = 2.5, ArH), 7.43 (t, 1H, *J* = 4, ArH), 7.54 (m, 2H, *J* = 4, ArH), 7.60 (m, 1H, *J* = 3, ArH), 7.63 (m, 2H, *J* = 5, ArH), 7.66 (s, 1H, ArH). ^13^C-NMR (DMSO-d_6_): δ 30.93, 34.64, 36.20, 123.10, 123.59, 124.28, 124.30, 125.27, 125.58, 125.61, 125.64, 126.83, 127.69, 127.84, 129.11, 129.37, 131.56, 133.24, 133.98, 135.29, 139.17, 150.92, 152.84, 154.46. MS analysis for C_26_H_23_ClF_3_N_3_S: Calcd mass: 501.13, found (*m*/*z*, ES+): 502.25.

*3-(4-tert-Butylphenyl)-5-(3-trifluoromethylbenzylsulfanyl)-4-(4-trifluoromethylphenyl)-4H-[1,2,4]triazole* (**UoST4**). Yield 70%, mp: 224–226 °C, ^1^H-NMR (DMSO-d_6_): δ 1.24 (s, 9H, *tert*-butyl), 4.43 (s, 2H, CH_2_), 7.26 (dd, 2H, *J* = 7.5, ArH), 7.39 (dd, 2H, *J* = 7.5, ArH), 7.48 (d, 2H, *J* = 9, ArH), 7.53 (m, 1H, *J* = 8.5, ArH), 7.62 (m, 2H, *J* = 8, ArH), 7.65 (s, 1H, ArH), 7.87 (d, 2H, *J* = 8.5, ArH). ^13^C-NMR (DMSO-d_6_): δ 30.827, 34.54, 36.12, 123.44, 124.156, 124.18, 125.56, 126.99, 127.02, 127.65, 128.83, 128.98, 129.23, 129.49, 129.97, 130.22, 133.10, 137.50, 138.99, 150.64, 152.71, 154.33. MS analysis for C_27_H_23_F_6_N_3_S: Calcd mass: 535.15, found (*m*/*z*, ES+): 536.28.

*4-(3-Bromophenyl)-3-(4-isopropylphenyl)-5-(3-trifluoromethylbenzylsulfanyl)-4H-[1,2,4]triazole* (**UoST5**). Yield 60%, mp: 201–203 °C, ^1^H-NMR (DMSO-d_6_): δ 1.15 (d, 6H, 2 CH_3_ of isopropyl), 2.84 (m, 1H, CH of isopropyl), 4.5 (s, 2H, CH_2_), 7.24 (m, 5H, *J* = 9.5, ArH), 7.43 (t, 1H, *J* = 8, ArH), 7.54 (t, 1H, *J* = 9, ArH), 7.57 (t, 1H, *J* = 7.5, ArH), 7.62 (d, 2H, *J* = 10, ArH), 7.67 (s, 1H, ArH), 7.73 (dd, 1H, *J* = 8, ArH). ^13^C-NMR (DMSO-d_6_): δ 23.51, 33.19, 36.05, 122.03, 123.00, 123.98, 124.11, 125.49, 126.63, 127.08, 127.87, 127.89, 129.21, 129.52, 130.60, 133.16, 135.28, 139.09, 150.03, 151.11, 154.40. MS analysis for C_25_H_21_BrF_3_N_3_S: Calcd mass: 532.04, found (*m*/*z*, ES+): 532.01

*3-(4-tert-Butylphenyl)-4-(4-methoxyphenyl)-5-(3-trifluoromethylbenzylsulfanyl)-4H-[1,2,4]triazole* (**UoST6**). Yield 47%, mp: 204–206 °C, ^1^H-NMR (DMSO-d_6_): δ 1.21 (s, 9H, *tert*-butyl), 3.79 (s, 3H, OCH_3_), 4.44 (s, 2H, CH_2_), 7.02 (d, 2H, *J* = 9, ArH), 7.13 (d, 2H, *J* = 8.5, ArH), 7.29 (d, 2H, *J* = 8.5, ArH), 7.36 (d, 2H, *J* = 8.5, ArH), 7.55 (t, 1H, *J* = 8, ArH), 7.65 (m, 3H, *J* = 11, ArH). ^13^C-NMR (DMSO-d_6_): δ 30.02, 32.54, 36.12, 55.52, 123.14, 124.56, 124.86, 126.56, 126.99, 127.22, 127.75, 128.23, 129.00, 129.23, 129.54, 129.67, 131.22, 132.10, 136.45, 139.39, 150.64, 151.72, 154.33. MS analysis for C_27_H_26_F_3_N_3_OS: Calcd mass: 497.17, found (*m*/*z*, ES+): 498.30.

*3-(4-tert-Butylphenyl)-4-p-tolyl-5-(3-trifluoromethylbenzylsulfanyl)-4H-[1,2,4]triazole* (**UoST7**). Yield 47%, mp: 184–186 °C, ^1^H-NMR (DMSO-d_6_): δ 1.22 (s, 9H, *tert*-butyl), 2.36 (s, 3H, CH_3_), 4.45 (s, 2H, CH_2_), 7.11 (d, 2H, *J* = 6.5, ArH), 7.29 (m, 4H, *J* = 9, ArH), 7.36 (d, 2H, *J* = 4.5, ArH), 7.54 (t, 1H, *J* = 7.5, ArH), 7.63 (m, 2H, *J* = 8.5, ArH), 7.68 (s, 1H, ArH). ^13^C-NMR (DMSO-d_6_): δ 20.86, 30.96, 34.62, 35.79, 123.13, 123.87, 124.24, 124.26, 125.57, 125.61, 125.64, 127.49, 127.55, 128.83, 129.09, 129.34, 129.63, 130.52, 131.38, 133.27, 139.97, 151.29, 152.64, 154.51. MS analysis for C_27_H_26_F_3_N_3_S: Calcd mass: 481.18, found (*m*/*z*, ES+): 482.22.

*3-(4-Isopropylphenyl)-4-(4-methoxyphenyl)-5-(3-trifluoromethylbenzylsulfanyl)-4H-[1,2,4]triazole* (**UoST8**). Yield 47%, mp: 214–216 °C,^1^H-NMR (DMSO-d_6_): δ 1.13 (d, 6H, 2 CH_3_ of isopropyl), 2.85 (m, 1H, CH of isopropyl), 3.75 (s, 3H, OCH_3_), 4.45 (s, 2H, CH_2_), 6.74 (dd, 1H, *J* = 8.5, ArH), 6.90 (t, 1H, *J* = 6.5, ArH), 7.09 (dd, 1H, *J* = 6.5, ArH), 7.21 (d, 2H, *J* = 5, ArH), 7.29 (d, 2H, *J* = 5, ArH), 7.38 (t, 1H, *J* = 7.5, ArH), 7.54 (t, 1H, *J* = 7.5, ArH), 7.61 (d, 1H, *J* = 8.5, ArH), 7.67 (d, 1H, *J* = 7, ArH), 7.72 (s, 1H, ArH).^13^C-NMR (DMSO-d_6_): δ 23.51, 33.18, 35.66, 55.52, 113.61, 115.44, 119.58, 124.08, 124.11, 125.52, 125.55, 126.52, 127.69, 128.95, 129.20, 129.45, 130.69, 133.15, 134.91, 139.13, 150.20, 154.35, 159.94. MS analysis for C_26_H_24_F_3_N_3_OS: Calcd mass: 483.16, found (*m*/*z*, ES+): 484.23.

*3-(4-Isopropylphenyl)-4-(4-fluorophenyl)-5-(3-trifluoromethylbenzylsulfanyl)-4H-[1,2,4]triazole* (**UoST10**). Yield 56%, mp: 203–205 °C, ^1^H-NMR (DMSO-d_6_ ): δ 1.23 (d, 6H, 2 CH_3_ of isopropyl), 2.97 (m, 1H, CH of isopropyl), 4.95 (s, 2H, CH_2_), 7.23 (d, 2H, *J* = 8.5, ArH), 7.38 (m, 3H, *J* = 10, ArH), 7.54 (d, 2H, *J* = 7.5, ArH), 7.67 (d, 2H, *J* = 8, ArH), 7.72 (d, 1H, *J* = 5, ArH), 7.87 (d, 2H, *J* = 5, ArH).^13^C-NMR (DMSO-d_6_): δ 23.02, 33.19, 36.04, 122.52, 123.88, 124.41, 125.45, 125.58, 125.66, 126.89, 127.88, 128.13, 129.19, 129.44, 130.14, 132.89, 135.27, 139.17, 150.69, 151.05, 154.39. MS analysis for C_25_H_21_F_4_N_3_OS: Calcd mass: 471.14, found (*m*/*z*, ES+): 472.04

*3-(4-tert-Butylphenyl)-4-(4-fluorophenyl)-5-(3-trifluoromethylbenzylsulfanyl)-4H-[1,2,4]triazole* (**UoST11**). Yield 65%, mp: 223–225 °C, ^1^H-NMR (DMSO-d_6_ ): δ 1.23 (s, 9H, *tert*-butyl), 4.45 (s, 2H, CH_2_), 7.26 (d, 2H, *J* = 6.5, ArH), 7.34 (m, 6H, *J* = 9.5, ArH), 7.54 (t, 1H, *J* = 8, ArH), 7.63 (m, 2H, *J* = 7.5, ArH), 7.68 (s, 1H, ArH). ^13^C-NMR (DMSO-d_6_): δ 30.67, 34.51, 35.75, 116.79 116.98, 123.67, 124.11, 125.46, 125.51, 125.54, 127.49, 128.96, 129.20, 129.49, 130.13, 130.24, 133.16, 139.08, 151.08, 152.52, 161.38. MS analysis for C_26_H_23_F_4_N_3_S: Calcd mass: 485.18, found (*m*/*z*, ES+): 486.22.

### 4.3. Organisms and Growth Conditions

The fungal strains used in this study were *C. albicans* (SC5314) and *C. auris* clinical isolate (obtained from the Centers for Disease Control and Prevention (CDC), Atlanta, GA, USA, South Asian clade, bronchoalveolar lavage]. Each fungal strain was inoculated into sterile Yeast Potato Dextrose broth (YPD) and incubated for 24 h at 37 °C. 

### 4.4. Determination of the Antifungal Activity

The anti-*Candida* activities of **UoST1**–**11** compounds and the **UoST5**-loaded NPs were measured on agar plates and liquid broth media according to modified Clinical and Laboratory Standards Institute (CLSI) and as described by Soliman et al. [12,37]. Briefly, 0.1 mL containing 10^4^ CFU/mL was spread on Sabouraud dextrose agar (SDA) plates. The plates were then incubated at 37 °C for 24 h with filter discs (8 mm diameter) saturated with different dilutions of the compounds (1–100 μg/mL). The inhibition zones (mm) were measured by determining the diameter of clear area. Similarly, the activity in liquid media was measured by incubating the aforementioned concentrations of the compounds into YPD broth media inoculated with 10^4^ CFU/mL in 96-well microplates at 37 °C for 48 h. The turbidity representing the microbial growth was measured by microplate reader (LT-4500, Labtech, Pocklington, York, UK) at OD_600_. Each test was performed in triplicate. Amphotricin-B (Cat#46006, Sigma-Aldrich) was employed as positive control. Cultures containing drug-free NPs, and cultures containing YPD broth media without the compounds or antimicrobials were employed as negative controls. 

### 4.5. Preparation of UoST5-Loaded NPs

Polymeric NPs were prepared by single emulsion/evaporation method [26]. Fifty µg of PLGA and 5 µg of **UoST5** were mixed in 2 mL of DCM. The organic solution was then added to 20 mL aqueous solution containing 2.5% PVA and emulsified by sonication for three successive cycles of 1 min/cycle at 30% voltage efficiency over an ice bath. The formed emulsion was stirred at 1000 rpm for 1 h at room temperature under vacuum until complete evaporation of DCM. The produced NPs were separated by centrifugation at 13,000 rpm for 40 min. The NPs were washed twice by chilled water and stored at −20 °C.

### 4.6. Characterization of UoST5-Loaded NPs 

#### Entrapment Efficiency

For the appropriate determination of the entrapment efficiency of the drug (UoST5), an efficient assay was developed based on the UV/VIS spectrophotometery absorbance of the drug. The maximum absorbance (ƛ_max_) of UoST5 was determined by UV/VIS spectrophotometer (SYNERGY H1, Biotek Ltd, Winoosk, VT, USA) and the selected ƛ_max_ (350 nm) was validated based on the selectivity, linearity and quantification limit. A calibration curve was developed for UoST5 in DCM (Supplementary Figure S1). The prepared NPs were mixed with DCM in order to completely dissolve the entrapped drug. The amount of entrapped drug was measured at 350 nm and the percentage of entrapment efficiency was calculated using the following formula (Equation (1)):

Entrapment efficiency % = (Amount of drug entrapped/Total amount of drug used) × 100(1)

### 4.7. Topology and Particle Size Measurements

The morphological characteristics of the UoST5-loaded NPs were evaluated using a scanning electron microscope (JSM-633OF; JEOL Ltd, Tokyo, Japan). NPs samples were suspended in distilled water and a drop of the solution was placed on a clean slide cover and left to dry under vacuum. The dried sample was mounted on carbon tape and sputter-coated with gold. The gold-coated samples were scanned and photomicrographs were taken at an acceleration voltage of 3 kV. The effective diameter, the size distribution, and zeta-potential of the developed NPs were measured by a Zetasizer (Malvern, Cambridge, UK). NPs were diluted with distilled water and the effective diameter and polydispersity index were determined using the Dynamic Light Scattering (DLS) mode at 25 °C. The Laser Doppler Velocimetry (LDV) mode was employed to measure the zeta potential (mV). 

### 4.8. In-Vitro Drug Release Study

Certain weights of NPs equivalent to 2.1 mg of UoST5 were suspended in dialysis membrane (MWCO = 12 kDa). The membranes were then immersed in 10 mL release media consisting of 0.9% NaCl: isopropanol (85:15) followed by incubation in water bath at 37 °C with shaking at 50 rpm. Samples were taken at different time intervals and the concentrations of UoST5 were determined. The cumulative percentage release (%) was calculated according to the following formula (Equation (2)):

The cumulative percentage release (%) = [Volume of sample withdrawn (ml)/Bath volume (v)] × P (t − 1) + Pt(2)

where Pt = percentage release at time (t) and P (t − 1) = percentage release previous to t.

### 4.9. Cell Toxicity Assay Using MTT Staining

The cell viability was performed using 3-(4,5-dimethylthiazolyl-2)-2,5-diphenyltetrazolium bromide (MTT) assay as described before [38]. Briefly, 96-well micro-plates were seeded with normal fibroblast cells (F180) (4000 cells per well) for 24 h. The best candidates **UoST5**, **7**, **8**, **11** at 3 µg/mL and **UoST5-NPs** at 3, 6.25, 12.5 µg/mL were suspended in DMEM media and filter sterilized prior to application on seeded cells. The color change was measured using a Multiskan Go instrument (Thermo Fisher Scientific, Japan) at 570 nm. Each experiment was repeated six times. Cell viability was calculated, using the following formula (Equation (3)):

% of living cells = (OD experimental)/(OD control) × 100(3)

where % of cell death was calculated by subtracting the living cells from the total number of cells [39].

### 4.10. Statistical Analysis

The data was collected and graphed using GraphPad Prism (5.04, GraphPad Inc., La Jolla, CA, USA). The data were analyzed by two-way analysis of variance (ANOVA) using Bonferroni’s Multiple Comparison Test. A *P*-value < 0.05 was considered as significant.

## 5. Conclusions

In conclusion, we have developed some novel azole derivatives by replacing the toxic aldehyde group of cuminaldehyde, a natural essential oil, with 1,2,4-triazole rings and the introduction of a 3-trifluoromethylbenzylsulfanyl moiety to selectively enhance the antifungal activity and reduce the toxicity. The anti-*Candida* activity of the best candidate **UoST5** was enhanced and prolonged by encapsulation in polymeric NPs. The formulated drug showed significant anti-*Candida* activities against *C. albicans* and *C. auris*, known multidrug-resistant human pathogens. The superior and prolonged activities were proposed to be due to the nature of the polymer employed since it can afford favorable adherence characteristics to *Candida* cells and hence can provide direct contact while establishing continuous release. 

## Figures and Tables

**Table 1:** MIC_50_ of UoST compounds against *C. auris*.

Compound	MIC_50_ (µg/mL)
UOST 5	2
UOST 7	5
UOST 8	15
UOST 11	3
Amphotericin B	0.3

## Supplementary Materials

The following are available online. Supplementary Figure S1 and spectroscopy date of compound **UoST5** including mass, ^1^H-NMR and ^13^C-NMR spectra.
