# Supplementary material for: Essential Oil-Based Design and Development of Novel Anti-Candida Azoles Formulation
Source: Molecules. 2020 Mar 24;25(6):1463. doi: 10.3390/molecules25061463 (PMC7146627; doi:10.3390/molecules25061463)
Supplement: Supplementary file 1 [file molecules-25-01463-s001.pdf]

molecules

Supplementary Materials: Essential Oil-Based Design and Development of Novel Anti-*Candida* Azoles Formulation

Rania Hamdy, Bahgat Fayed, Alshaimaa M. Hamoda, Mutasem Rawas-Qalaji ,Mohamed Haider and Sameh S. M. Soliman

Supplementary Figure 1

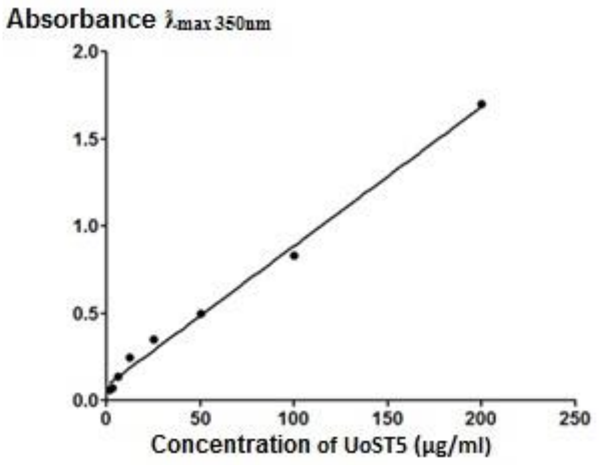

Supplementary Figure 1. Calibration curve for the dilution series of UoST5 at $\lambda_{max\ 350}$ showing linearity over concentration range of 3.125-200 µg/ml .The maximum absorbance (λ_{max}) was determined by UV/VIS spectrophotometer then serial dilution of UoST5 in DCM was prepared and measured at λ_{max} using DCM as sample blank.

Supplementary file 1. Spectroscopic identification of UoST5

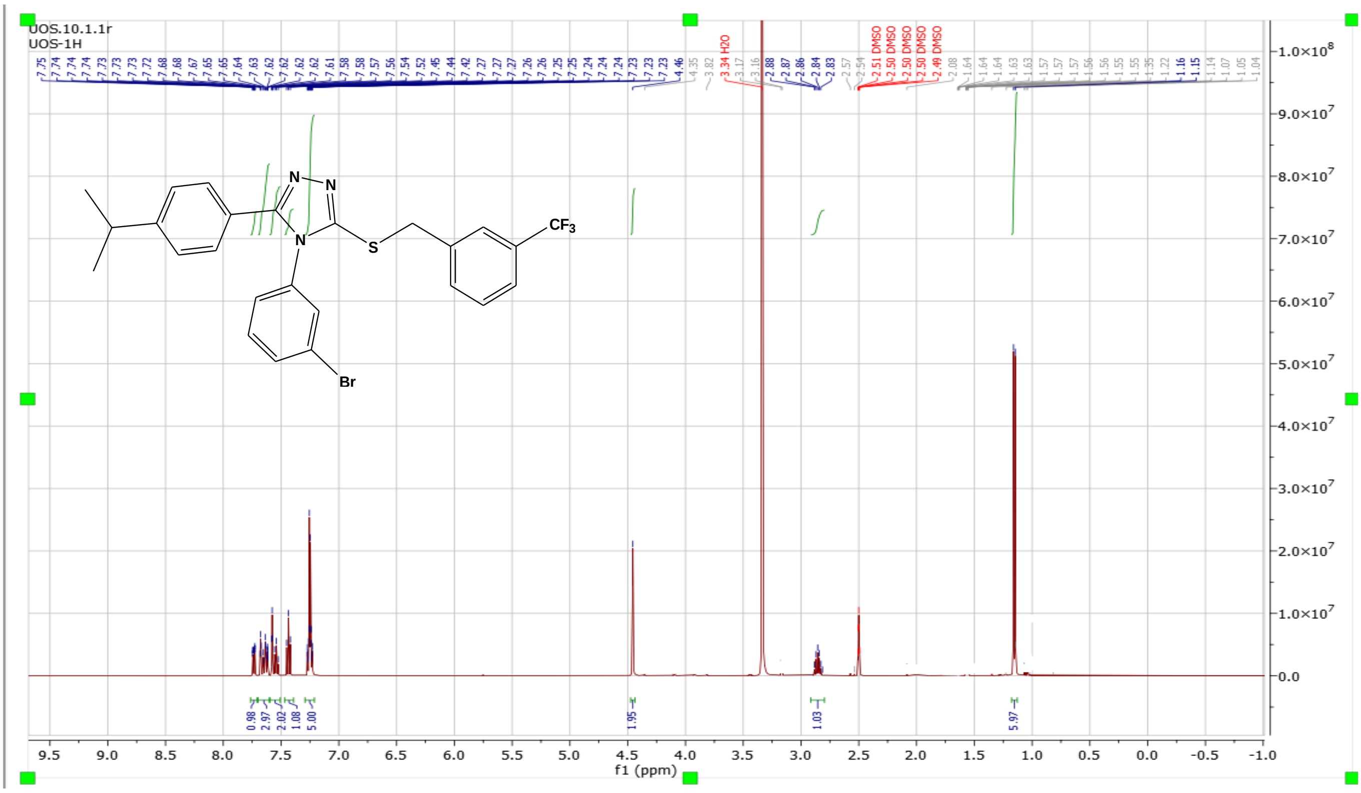

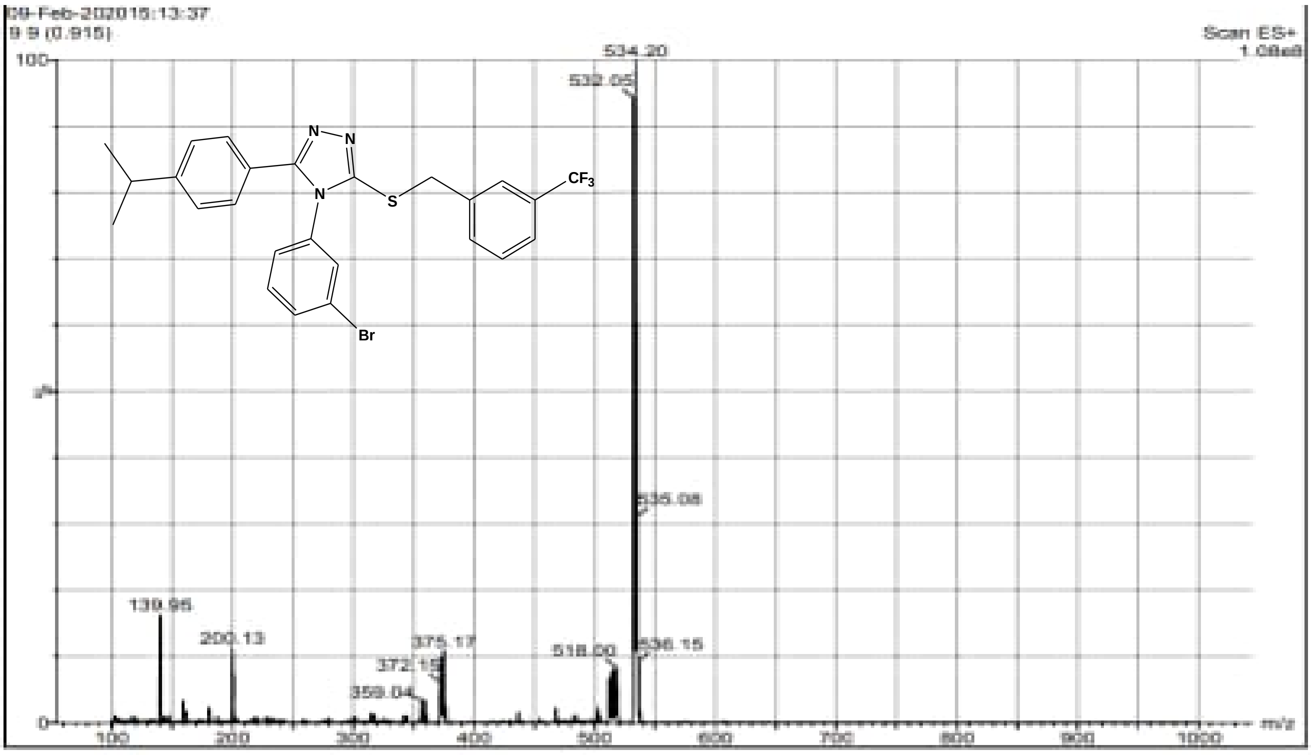
09-Feb-202015:13:37
9.9 (0.915)
Scan ES+
1.08e8
100
%
0
534.20
532.05
535.08
139.95
200.13
375.17
372.15
359.04
518.00
536.15
100
200
300
400
500
600
700
800
900
1000
m/z
N
N
N
S
CF3
Br

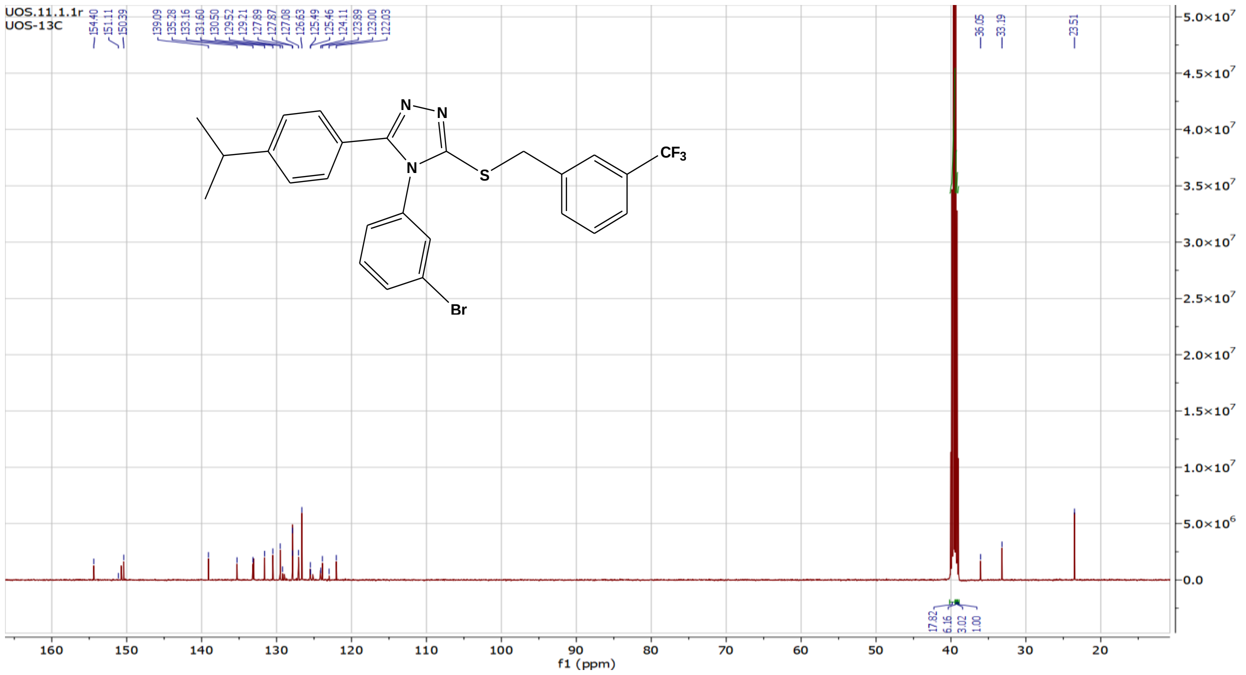
UOS.11.1.1r
UOS-13C
154.40
151.11
150.39
139.09
135.28
133.16
131.60
130.50
129.52
129.21
127.89
127.87
127.08
126.63
125.49
125.46
124.11
123.89
123.00
122.03
36.05
33.19
23.51
N
N
N
S
CF_3
Br
17.82
6.16
3.02
1.00
f1 (ppm)
